# Metagenomic Analysis of Microbial Composition Revealed Cross-Contamination Pathway of Bacteria at a Foodservice Facility

**DOI:** 10.3389/fmicb.2021.636329

**Published:** 2021-04-12

**Authors:** Eun Seob Lim, Jin Ju Kim, Woo Jun Sul, Joo-Sung Kim, Bomin Kim, Hun Kim, Ok Kyung Koo

**Affiliations:** ^1^Department of Food Biotechnology, Korea University of Science and Technology, Daejeon, South Korea; ^2^Food Safety Research Team, Korea Food Research Institute, Wanju-gun, South Korea; ^3^Department of Systems Biotechnology, Chung-Ang University, Anseong, South Korea; ^4^Department of Medicinal Chemistry and Pharmacology, University of Science and Technology, Daejeon, South Korea; ^5^Center for Eco-Friendly New Materials, Korea Research Institute of Chemical Technology, Daejeon, South Korea; ^6^Department of Food and Nutrition, Gyeongsang National University, Jinju, South Korea; ^7^Institute of Agriculture and Life Science, Gyeongsang National University, Jinju, South Korea

**Keywords:** microbial diversity, biogeography, metagenome, cross-contamination, foodservice facility

## Abstract

Bacterial contamination of food-contact surfaces can be a potential risk factor for food quality and safety. To evaluate the spatial and temporal variations of the potential cross-contamination routes, we conducted a biogeographical assessment of bacteria in a foodservice facility based on the diversity of microflora on each surface. To this end, we performed high-throughput amplicon sequencing of 13 food-contact and non-food contact surfaces in a foodservice facility throughout a year. The results showed that *Bacillus*, *Acinetobacter, Streptophyta*, *Enterobacter*, *Pseudomonas*, *Serratia*, *Enhydrobacter*, *Staphylococcus*, *Paracoccus*, and *Lysinibacillus* were the dominant genera found on the kitchen surfaces of the foodservice facility. Depending on the season, changes in *Firmicute/Proteobacteria* ratios were observed, and the fan becomes the main source of outdoor air contamination. The microbial flow associated with spoilage was also observed throughout food preparation. Taken together, our results would be a powerful reference to hygiene managers for improvement of food processes.

## Introduction

Most foodborne illness outbreaks take place in foodservice facilities, such as hospitals, educational institutions, workplace cafeterias, restaurants, and other establishments. An average of 620 outbreaks was reported every year in restaurants (56% of total outbreaks) between 1998 and 2013 in the United States (Angelo et al., 2017). In general, microbes from food workers and raw ingredients are considered the major contributors to contamination, which can lead to cross-contamination and large outbreaks. In the EU, it has been reported that salmonellosis, a common bacterial disease affecting the intestinal tract, is related to cross-contamination between raw/cooked food and food-contact surfaces (Osimani et al., 2016). Microorganisms in the food manufacturing environment contain spoilage and/or pathogenic microbes that can cause quality issue and serious health problems by cross-contamination during improper handling of raw material with poor hygiene (Egan et al., 2007; Oliveira et al., 2014). Therefore, understanding the contamination path is the primary step for the safety control.

Restaurants and catering facilities are readily exposed to a diverse microbiota derived from raw ingredients, food workers, and food processing environments (Stellato et al., 2015b). When the microorganisms are introduced into foodservice facilities, food-contact surfaces are good environments for microbial colonization and persistence (De Filippis et al., 2021). Surface-attached microorganisms form biofilms and generate protective substances to survive extreme environmental conditions, such as dehydration, temperature, pH, and antimicrobial treatments (Bridier et al., 2015; Flemming et al., 2016). The biofilm not only protects microorganisms from sanitizing conditions but also readily transfers microorganisms to food or other food-contact surfaces, where the microbes detach from the biofilm and find new niches to survive the starving environment (Stoodley et al., 2002). Food or food handlers provide an ideal vehicle for the microorganisms (González-Rivas et al., 2018). Furthermore, it has been reported that the bacteria isolated from a food service facility after cleaning and disinfection exhibited a strong ability for biofilm formation compared to the standard isolates (Lim et al., 2017). The results of the aforementioned study suggest that the cleaning and disinfection process provides selection pressure for the bacterial strains containing strong ability for biofilm formation. Given that the presence of microbes and their flow can contribute to cross-contamination and give rise to serious food safety concerns, analysis of microbial communities is required for a better understanding of foodborne outbreaks (Suslow, 2001).

The development of high throughput sequencing technology made it possible to analyze the taxonomic diversity of various environmental microbial communities with large-scale sequencing data (Aravindraja et al., 2013). Microbial community analyses have thus been extensively carried out with foods (Leonard et al., 2015; Escobar-zepeda et al., 2016), processing facilities (Bokulich et al., 2015; Stellato et al., 2016; Falardeau et al., 2019), and their environment (Kembel et al., 2012; Dunn et al., 2013). However, most cases focused on the specific type of food industry related to the microbes from the major food ingredients. There are still limited information related with the microbial flow on the surfaces of food service facilities contaminated from a wide range of sources such as restaurants or cafeterias. Considering that food workers and raw ingredients are the major contributors to contamination in the kitchen environment, the surfaces of foodservice facilities have been underestimated as a possible source of microbial contamination. In this study, we analyzed the sequences of 16S rRNA genes by next-generation sequencing to investigate the diversity of the microflora in a foodservice facility. By categorizing the microbial configuration according to various areas and different time points, we explored the potential risks of contamination and the potential microbial flow during the cooking process in a foodservice facility.

## Materials and Methods

### Sample Collection From the Foodservice Facility

**FIGURE 1:**
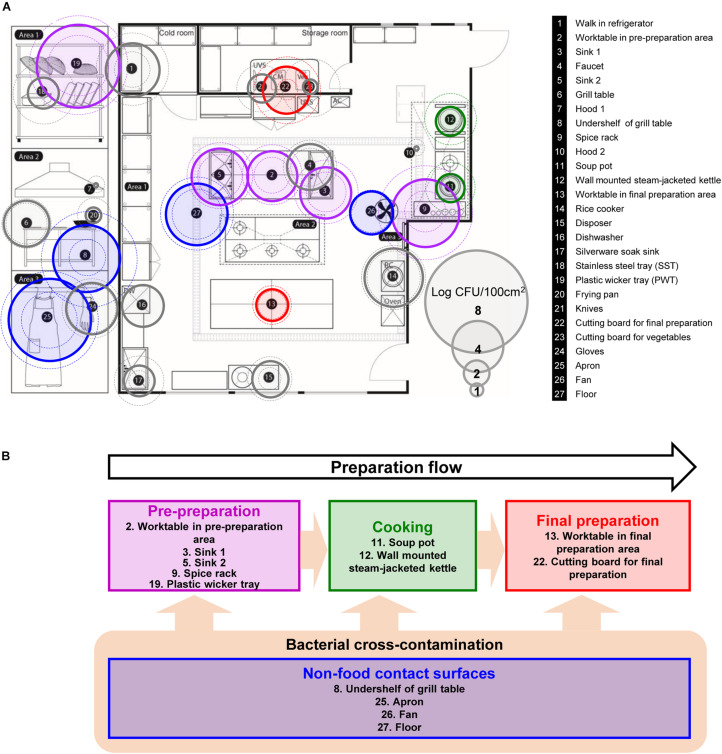
Selected food-contact and non-food contact surfaces, and the preparation flow of the kitchen in a foodservice facility. **(A)** Total aerobic count (TAC) of twenty-seven surfaces, where each surface is indicated with number with information on the right, **(B)** schematic diagram of the preparation flow with selected surfaces in the kitchen. The colored circles with solid line are the average TAC of each surface. Outer dotted circles are the maximum counts and inner dotted circles are minimum counts of TAC. Circles that are colored in purple, green, red, and blue indicate each cooking process as pre-preparation area, cooking, final preparation area, and cooking environment, respectively. DW, dishwasher; UVS, UV sterilizer; Veg, vegetable cutting board; AC, air conditioner; RC, rice cooker.

A kitchen in a foodservice facility in Seongnam-si, Gyeonggi-do, Republic of Korea with an average daily attendance of over 250 people was selected for sampling the native microflora. Twenty-seven surfaces (100 cm^2^/each surface) exposed to food directly or indirectly were selected for the assessment of the microbial contamination in the kitchen of a foodservice facility (Figure 1A). Each surface was categorized into four different surface types based on the preparation flow; pre-preparation area, cooking area, final preparation area, and non-food contact surfaces (Figure 1B). Each surface was defined using a sterile stainless-steel frame, and then each sample was collected by vigorous swabbing with a 3M Pipette Swab Plus in 10 mL buffered peptone water broth (BPW) (3M Korea, Seoul, Republic of Korea) ten times vertically, horizontally, and diagonally within the frame followed by the MFDS guideline (Ministry of Food and Drug Safety, MFDS, 2020). All samples were taken after routine daily cleaning and disinfection of the facility. Each sampling was conducted quarterly over 1 year between Dec 2014 (Q1) and Oct 2015 (Q4). After sampling, the swabs in BPW were vortexed for 1 min, and the resuspended cells were plated onto Plate Count Agar (BD Difco, Sparks, MD, United States) with up to a dilution of 10^–7^ and incubated at 30°C for 48 h for quantification. Sterile latex gloves were worn during collection to minimize any cross-contamination from the researcher’s hands.

### DNA Extraction and Barcoded Pyrosequencing

For the biogeographical analysis, thirteen contaminated areas were selected for the 16S rRNA gene analysis using high-throughput amplicon sequencing. Metagenomic DNA of each sample was extracted using a MasterPure Gram Positive DNA Purification Kit (Epicentre, Madison, WI, United States) according to the manufacturer’s instructions, and the DNA concentration was measured using NanoVue (GE Healthcare, Buckinghamshire, United Kingdom). Extracted DNA was used for PCR amplification using primers targeting the V1–V3 region of the 16S rRNA gene (Ercolini et al., 2012). PCR primers were designed as follows: 5′-[Adapter]-[Key]-[Barcode]-[Linker]-[16S rRNA universal primer]-3′. The barcode was only used for 518R primer. The primer sequences are as follows: 27F (5′-CCTATCCCCTGTGTGCCTTGGCAGTCTCAGACGAGTTTG ATCMTGGCTCAG-3′) and 518R (5′-CCATCTCATCCCTG CGTGTCTCCGACTCAG-[Barcode]-ACWTTACCGCGGCTG CT GG-3′; the barcode was a 7-11 bp long unique sequence). The PCR conditions used for the amplification were as follows: initial denaturation at 95°C for 5 min; 30 cycles of denaturation at 95°C for 30 s; annealing at 55°C for 30 s; and elongation at 72°C for 30 s; with a final extension at 72°C for 7 min. The amplified products were purified using resin columns, and sequencing was performed by ChunLab, Inc. (Seoul, Korea), using a Roche 454 GS-FLX+ (Roche, CT, United States) in accordance with the manufacturer’s instructions.

### Pyrosequencing Data Analysis

The pyrosequencing reads were filtered to remove low-quality reads (average quality score <25 bp and reads <200 bp), which were sorted using a barcode and de-noised with QIIME (Quantitative Insights into Microbial Ecology) (v.1.8.0) using the script *split_libraries.py* and *denoise_wrapper.py* (Caporaso et al., 2010). The filtered reads were clustered into the operational taxonomic units (OTUs) with *pick_otus.py* at a sequence identity of 97% by UCLUST (Edgar, 2010). Representative sequences from each OTU were assigned to a taxonomy by the RDP (Ribosomal Database Project-II) Classifier using a 50% confidence threshold (Wang et al., 2007) using SILVA database (Yilmaz et al., 2014), aligned with PyNAST aligner (Caporaso et al., 2010), and used to construct a phylogenetic tree using FastTree algorithm (Price et al., 2010) in QIIME. Since there were no significant differences in data before and after chimera removal, chimeras were not removed. The DDBJ Sequence Read Archive number for the 16s rRNA sequences was reported as DRA006215.

### Analysis of Genetic Diversity

Chao1, phylogenetic diversity, and Simpson and Shannon diversity indexes were analyzed for the genetic diversity of each sample using the QIIME script *alpha_diversity.py.* Unweighted Unifrac distance matrices (Lozupone and Knight, 2005) were calculated with the QIIME script *beta_diversity.py* using the phylogenetic tree. UPGMA hierarchical clustering was performed, and clustering dendrograms were conducted based on the unweighted method. To identify the differences in bacterial community compositions among the samples, a principal coordinate analysis (PCoA) based on the unweighted UniFrac distance matrices was performed with *principal_coordinates.py*. A linear discriminant of the effect size estimation (LEfSe) (Segata et al., 2011) was used to identify OTUs with significantly different abundances between samples. The linear discriminant analysis (LDA) effect size (LEfSe) of the algorithms for the distinctive features was 3.5, and a one-against-all comparison was performed.

### Microbial Biogeographic Analysis

To estimate the microbial cross-contamination ratio and potential biogeographical flow during the cooking process, we used Bayesian methods based SourceTracker (Knights et al., 2011). For the analysis, we estimated the potential microbial contamination from various sources, and the microorganisms from each source were selected based on the previous literature. The numbers of microorganisms from each source were 246 in hand, and 54 in oral (Costello et al., 2009), 4 in outdoor air (Kembel et al., 2012), 89 in soil (Lauber et al., 2009), and 107 in phyllosphere (Redford et al., 2010).

### Statistical Analysis

A LDA was used to identify OTUs with significantly different abundances between samples. The alpha values for both the Kruskal-Wallis and pairwise Wilcoxon rank-sum tests were 0.05. Alpha diversities were evaluated with Tukey Honestly Significantly Difference (HSD) tests with a significance level of 0.05. PCoA among the beta diversity was performed with *principal_coordinates.py* based on the unweighted UniFrac distance matrices. For microbial cross-contamination and potential biogeographical flow analysis, the Bayesian method based SourceTracker was used.

## Results

### Microbial Contamination in the Kitchen

The total aerobic count of each 100 cm^2^ surface is presented in Figure 1A. Of 27 surfaces, undershelf of the grill table, the spice rack, the plastic wicker tray, and the apron were contaminated with more than 5 Log CFU/100 cm^2^. The plastic wicker tray and apron were the most contaminated surfaces, with average values of 6.49 and 6.41 Log CFU/100 cm^2^, respectively. The hoods were particularly low, with an average of 0.33 Log CFU/100 cm^2^. Despite the low temperature condition, shelves in the walk-in refrigerator contained relatively high bacterial counts of 4.20 Log CFU/100 cm^2^. Food contact surfaces and non-food contact surfaces didn’t show any significant differences by the average of 3.29 ± 1.53 and 3.37 ± 1.44 Log CFU/100 cm^2^, respectively. When compared by the sampling time (quarter), there were no significant differences between each quarter with 3.31 ± 1.90, 3.16 ± 2.19, 3.33 ± 1.71, and 3.62 ± 1.94 Log CFU/100 cm^2^ from Q1 to Q4, respectively.

Based on the aerobic counts, a total of thirteen surfaces were selected for further pyrosequencing analysis, that were about 4 Log CFU/100 cm^2^ or above, located in each preparation area and are related to food-contact/non-food contact surface including personnel related surface, apron. Soup pot and frying pot were included to observe the contamination in cooking area even with lower aerobic counts as well as the worktable in final preparation area. The analyzed sequence information such as raw and filtered sequence information, denoised sequence number and others are summarized in Supplementary Table 1 and the rarefaction curve in Supplementary Figure 1.

### Alpha Diversity Analysis by Each Surface Type

**FIGURE 2:**
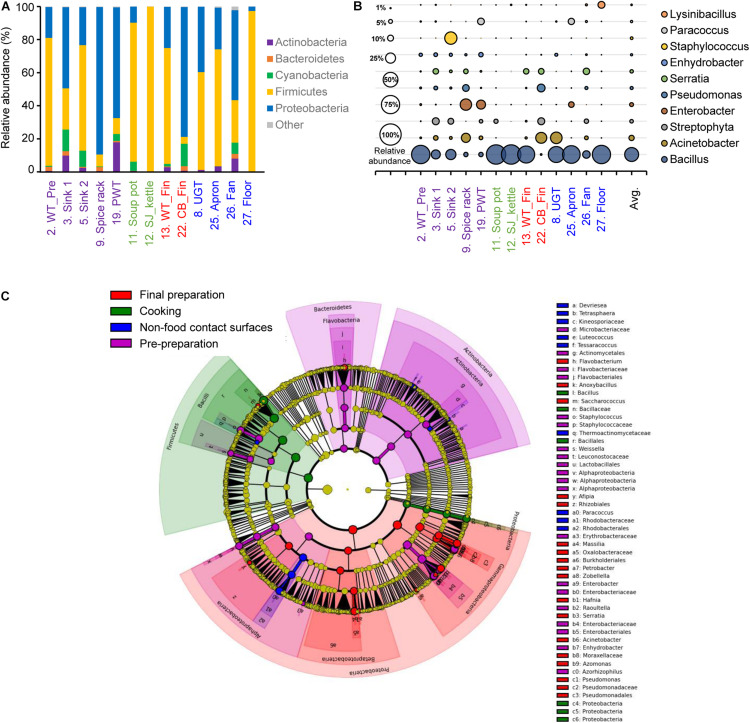
Comparison of the bacterial distribution in each food-contact and non-food contact surfaces. Relative abundances at phylum level **(A)** and genus level **(B)**, and the LEfSe cladogram **(C)** of taxonomic LDA score higher than 3.0. The pre-preparation area, cooking area, final preparation area, and the cooking environment indicated as purple, green, red, and blue, respectively.

The alpha diversity of each surface type showed that the least diverse microbial community was observed in the soup pot with a Chao1 of 61.82, followed by the steam-jacketed kettle with an average Chao1 of 138.48. The most diverse surface with a Chao1 of 973.30 was the fan, compared to the average of 364.06. Based on the Shannon diversity index comparison of each surface type, the pre-preparation area, final preparation area, and non-food contact surfaces showed no significant differences, whereas the cooking area had a significantly low diversity and richness compared to other surface types (Supplementary Figure 2). Relatively high abundance of the phyla Firmicutes and Proteobacteria was detected from all samples used in this study; in particular, Firmicutes were dominant in the cooking area and non-food contact surfaces at levels of 90.0 and 62.1%, respectively (Figure 2A and Supplementary Figure 2). At the genus level, *Bacillus* species were the most dominant genera, at 79.0 and 51.0%, in the cooking and non-food contact surfaces, respectively, whereas the *Bacillus* species was detected with levels of 27.4 and 24.8% in the final preparation and pre-preparation areas, respectively (Figure 2B). Proteobacteria accounted for 63.5% of total bacteria in the final preparation area and 54.6% in the pre-preparation area (Figure 2A). In the phylum Proteobacteria, genus Acinetobacter was dominant on the applicable surfaces: 15.7% in the final preparation area, 8.0% in the pre-preparation area, and 9.8% in the cooking area (Figure 2B).

The LEfSe of each surface type resulted in a significantly different microbial distribution (Figure 2C). The surfaces of the final preparation area exhibited a distinguished distribution of classes Gammaproteobacteria and Betaproteobacteria, and the genus Acinetobacter and Pseudomonas. The phyla Bacteroidetes and Actinobacteria significantly dominated in the pre-preparation area, whereas Bacillus in the phylum Firmicutes was significantly noted in the cooking area, including soup pots and steam-jacketed kettles. In particular, limited bacteria such as genera *Paracoccus* and *Luteococcus* in non-food contact surfaces mostly overlapped from food-contact surfaces.

### Alpha Diversity Analysis of Each Sampling Time

**FIGURE 3:**
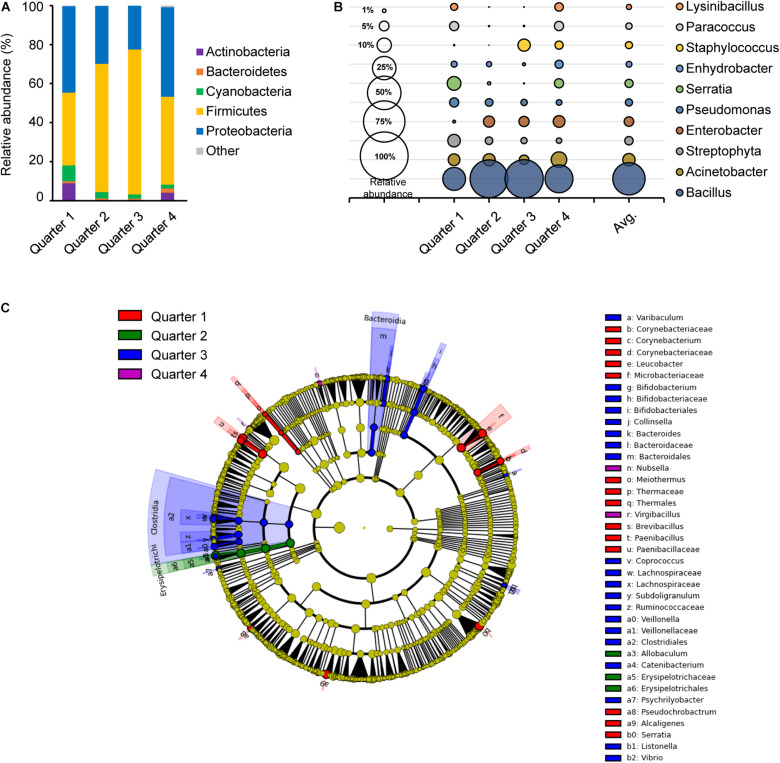
Comparison of the bacterial distribution in each sampling time point. Relative abundances at phylum level **(A)** and genus level **(B)**, and the LEfSe cladogram **(C)** of taxonomic LDA score higher than 3.0 by each sampling time point in 1 year; quarter 1, quarter 2, quarter 3, and quarter 4 are indicated as red, green, blue, and purple, respectively.

Sampling time in temperature and humidity can affect the abundance and the diversity of microorganisms in the environment and also results in different bacterial growth rates (Grassly and Fraser, 2006). Average temperatures and humidity on site during the sampling time were −1.85°C and 53.3% in Q1, 13.7°C and 70.0% in Q2, 23.0°C and 75.4% in Q3, and 19.4°C and 67.5% in Q4, respectively (data not shown). There was a consistent significant difference between Q1 and Q2 for their observed OTU, PD whole tree, Shannon and Simpson diversity indices, where Q1 was significantly higher than Q2 (Supplementary Figure 2). In Shannon and Simpson diversity indices, Q2 and Q4 were also significantly different. These results agreed to the bacterial distribution in Figure 3.

The core bacteria of each quarter were primarily composed of Firmicutes and/or Proteobacteria with a range of 35.7 to 68.8% and 29.6 to 50.0%, respectively (Figure 3A). Firmicutes were more abundant in Q2 and Q3, while Proteobacteria were more abundant in Q1 and Q4. Genus *Acinetobacter, Streptophyta*, *Pseudomonas* and *Staphylococcus* species were distributed in close proximity in each quarter (Figure 3B). However, *Bacillus* was higher with relative abundance in Q2 and Q3 with 0.63 and 0.65, respectively, while *Lysinibacillus*, *Paracoccus*, and *Serratia* were more abundant in Q1 and Q4. A taxonomic comparison in the LEfSe exhibited few distinguishable bacteria, such as the class *Clostridia* and *Bacteroidia* in Q3 (Figure 3C). *Clostridia, Bifidobacterium*, *Bacteroides*, and *Vibrio* in Q3 and *Leucobacter*, *Brevibacteriu*, *Corynebacterium*, and *Paenibacillus* in Q1 were prominently recognized, and *Allobaculum* in the class *Erysipelotrichi* was notably observed in Q2. Only two genus, *Nubsella* and *Virgibacillus* were distinctively observed in Q4.

### Beta Diversity

**FIGURE 4:**
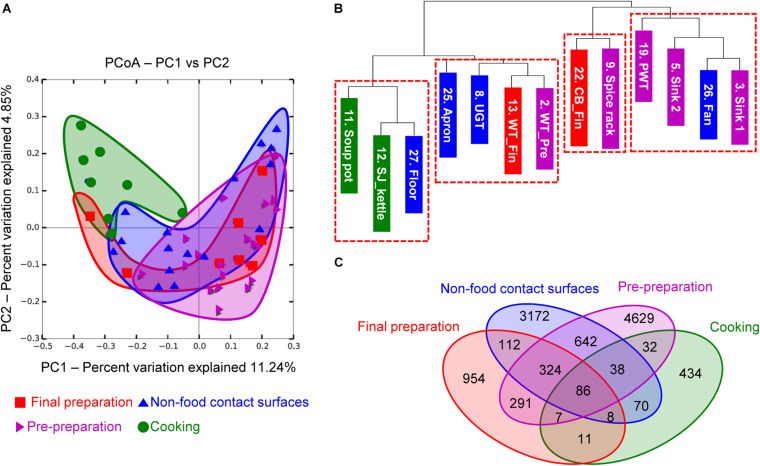
Principal coordinate analysis (PCoA) based on unweighted UniFrac distances **(A)**, unweighted dendrogram **(B)**, and the distribution of shared OTUs **(C)**. Purple, green, red, and blue indicate location of each cooking process as pre-preparation area, cooking area, final preparation area, and cooking environment, respectively.

To investigate the correlation between each surface, the microbial diversity was analyzed with beta diversity comparison. In the unweighted PCoA results, the cooking area showed a distinctively different distribution compared to other surface areas, while the pre-preparation area, final preparation area, and non-food contact surfaces overlapped with similar patterns of diversity (Figure 4A). The dendrogram analysis in Figure 4B also showed that the samples from the cooking area were not clustered with the samples of the surfaces from the pre-preparation area. In particular, the two sinks of the pre-preparation area were clustered with the fan, suggesting that the close proximity caused continual microbial transfer during the process (Figure 4B). The final preparation area shared 708 OTUs with the pre-preparation area, which was 39.5% of the total OTUs in the final preparation area, followed by 530 OTUs in the cooking environment, and 112 OTUs with the cooking area (Figure 4C). The final preparation area and pre-preparation area shared bacterial species such as *Streptophyta*, *Bacillus*, *Corynebacterium*, *Propionibacterium*, *Staphylococcus*, *Lactobacillus*, *Weissella*, *Lactococcus*, *Enterobacteriaceae*, *Acinetobacter*, and *Pseudomonas* spp., which is likely related to non-heated foods. *Enterobacteriaceae*, *Pseudomonas*, and *Staphylococcus* were commonly detected in pre-preparation, cooking, and final preparation, but not in the cooking environment (data not shown). All four types of surfaces shared 86 OTUs with *Bifidobacterium*, *Bacteroides*, *Streptophyta*, *Bacillus*, *Propionibacterium*, *Staphylococcus*, *Lactobacillus*, *Leuconostoc*, *Weissella*, *Lactococcus*, *Enterobacteriaceae*, *Acinetobacter*, and *Pseudomonas*, which were considered highly cross-contaminated strains in this study.

### Bayesian Approach for Cross-Contamination Analysis

**FIGURE 5:**
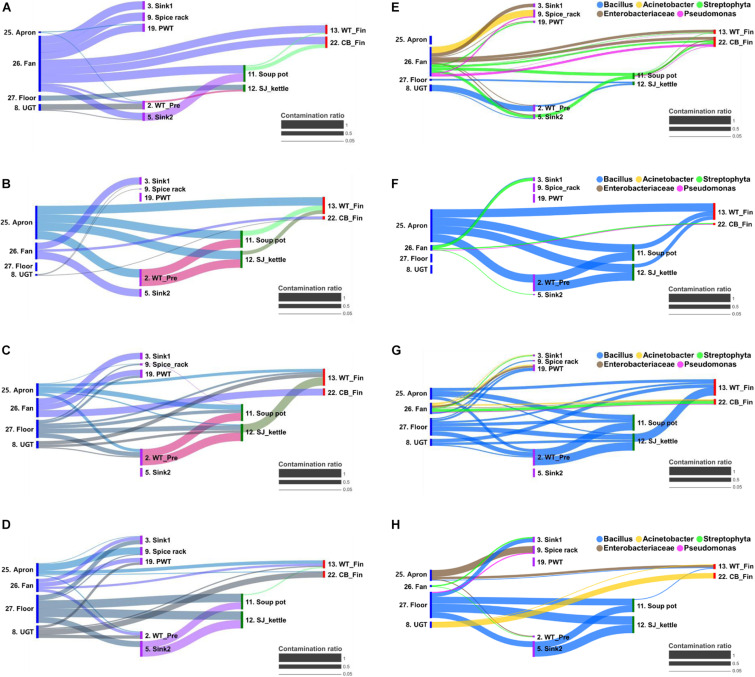
Bacterial transfer during food preparation represented by contamination ratio. The contamination ratio of overall bacteria **(A–D)** and five major bacterial genus **(E–H)** at quarter 1 **(A,E)**, 2 **(B,F)**, 3 **(C,G)**, and 4 **(D,H)**. The contamination ratio was calculated using the Bayesian-based SourceTracker based on the similarity of the microbial distribution between surfaces. CB_Fin, cutting board for final preparation; PWT, plastic wicker tray; SJ_kettle, steam-jacketed kettle; UGT, undershelf of the grill table; WT-Fin, worktable for final preparation; WT_Pre, worktable for pre-preparation.

The cross-contamination pathway is presented in Figure 5 for the bacterial transfer between surfaces in each quarter and the five major bacterial genus. When a high contamination ratio was considered to correspond with a high cross-contamination ratio, our results showed that the cross-contamination ratio between food-contact surfaces and non-food contact surfaces was more apparent than that within the food-contact surfaces (Figure 5). In particular, the fan was observed to be the strongest source of cross-contamination, because the fan was connected to all surface types, followed by aprons, the floor, and undershelf of the grill table. The apron was also found to be strongly connected to many types of food-contact surfaces in the pre-preparation, cooking, and final preparation areas (Figure 5). The result demonstrated the bacterial transfer from workers and the external environment to the meal during food preparation. Of five selected major bacterial genera *Bacillus, Acinetobacter, Streptophyta, Enterobacteriaceae*, and *Pseudomonas*, the genus *Bacillus* was detected as the main source of contribution on all surfaces through all quarters, except in Q1 (Figure 5).

### Bayesian Approach for Contamination Sources

**FIGURE 6:**
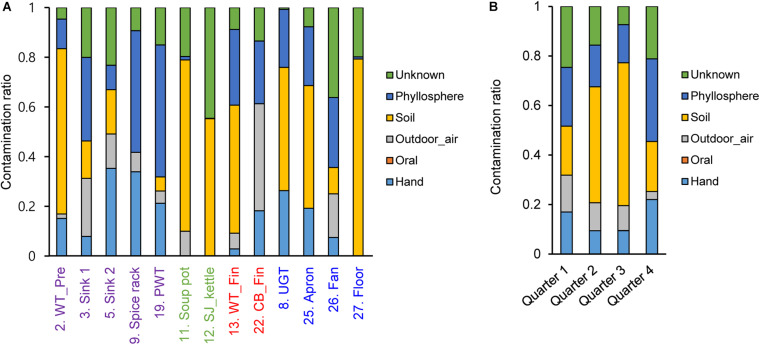
Contamination ratio comparison from different sources to each surface **(A)** and each sampling time point **(B)**. The pre-preparation area, cooking area, final preparation area, and the cooking environment indicated as purple, green, red, and blue, respectively.

To investigate contamination sources, five contamination sources (e.g., hand, oral, and soil) were analyzed by a Bayesian approach based on each surface and sampling time point. Our results showed that the contamination ratio varied by quarters and surfaces (Figure 6). In particular, bacterial contamination on the hand highly contributed to contamination of the apron, sink2, spice rack, worktable, and undershelf of the grill table, whereas oral bacteria were rarely observed (Figure 6). Airborne bacterial contamination was mostly detected in cutting board for final preparation, and rhizosphere bacterial communities dominantly presented in the floor, kettles, and soup pots. Samples derived from other surfaces such as the apron, worktables, and spreaders showed phyllosphere bacteria, which were primary observed in the plastic wicker tray and spice rack where fresh produce or plant based spices such as red pepper powder were located, suggesting that the surfaces were contaminated by soil (Figure 6). In terms of the contribution of bacterial species, we found that the main bacteria for hand contamination were *Acinetobacter*, *Staphylococcus*, *Pseudomonas*, and *Paracoccus*, whereas bacteria detected from outdoor air samples include *Streptophyta*, *Pseudomonas*, *Enterobacteriaceae*, *Burkholderia*, *Acinetobacter*, and *Janthinobacterium* (Supplementary Table 2). *Bacillus* and *Pseudomonas* were found to be the main contributors of soil contamination, and *Enterobacteriaceae*, *Acinetobacter*, *Bacillus*, and *Serratia* were derived from phyllosphere contamination.

## Discussion

Microbial community in each food processing plant or each kitchen may differ by various environmental factors. This study highlights whether there is a microbial community change in one kitchen by the sampling time or by the surface type such as food contact and non-food contact surfaces or the production layout. The temporal and spatial differences can affect the diversity of the microbial community in a food facility. In this study, the average viable bacterial concentrations of food-contact and non-food contact surfaces were not significantly different. United States Public Health Service recommends no more than 100 CFU/50 cm^2^ in food contact surface, and European Commission recommended <10 CFU/cm^2^ for cleaned and disinfected surfaces in meat establishments (European Commission Decision, 2001; Evancho et al., 2001). However, Korean government is still working on the guideline for aerobic count on food-contact surfaces, while zero tolerance is applied to *Salmonella* and coliform (Ministry Ministry of Food and Drug Safety, MFDS, 2020). Although the sampling condition may be different, when followed the United States guideline, numbers of surfaces are over the limit. While other food service facilities had also shown over 100 CFU/50 cm^2^ in previous studies, standardized sampling time, condition, and sampling methods should be followed to compare the condition. Nevertheless, extensive hygiene practice is recommended in this facility.

The effects of temperature and climate have been reported in microbial communities and the incidence rates of infectious diseases (Grassly and Fraser, 2006). For example, after extreme water-related weather events, including flooding and rainfall, the population of *Vibrio*, *Campylobacter*, *Leptospira*, *Cryptosporidium*, and norovirus increased in the range from 6 to 24% (Manfreda and Cesare, 2016). High specificity of feces-related genera such as *Bacteroides* and *Bifidobacterium* has also been reported, showing that the feces are vulnerable to contamination in the summer season (Lloyd-Price et al., 2016). Given that the microbial distribution was influenced by season, we investigated the microbial community according to the average atmospheric temperature (18.0 and 8.6°C). The overall bacterial concentration of the surfaces were not affected by the atmospheric temperature; however, the bacterial community composition changed by the season. Notably, human pathogens such as genera *Vibrio* was detected in Q3 and there was a significant increase in the relative abundance of *Staphylococcus* showing that a greater attention is recommended in hotter season, while other seasons showed only few bacteria to be recognized. In addition, two distinctive rate of *Bacillus* spp. and the Firmicute/Proteobacteria ratio were observed by the warmer or colder seasons (Supplementary Figure 2). Similarly, Djekic et al. (2016) reported that the climate condition in foodservice establishments affects the presence of hygiene indicators, such as *Enterobacteriaceae* and *Staphylococcus*.

With regard to the surface type, our research showed that highly specified bacteria are allocated with more accessible contamination of each surface; *Bacillus* in the cooking area, *Acinetobacter* and *Pseudomonas* in the final preparation area, and *Enterobacteriaceae* in the pre-preparation area. Overall, the most abundant bacterial species in this study was *Bacillus*, which can survive and persist in the environment for years by the formation of endospores. *Bacillus* spp. should be removed since they can germinate into vegetative cells causing spoilage or sporadic outbreaks (Giffel et al., 1995). This observation was similar with our previous finding that 34.3% of *Bacillus* spp. were isolated from food-contact surfaces in a cafeteria kitchen, and isolated *Bacillus* spp. were detected on 19 out of 23 surfaces in the kitchen (Lim et al., 2017). Considering that *Bacillus* has been reported to contaminate via dust and/or biofilms in the form of endospores (Faille et al., 2014), *Bacillus* detected in this study could be contaminated from soil and the phyllosphere in the food ingredients to the apron, floor, and undershelf of the grill table, which can lead to contamination of the worktable in the final preparation area or affect the final food products through the pre-preparation work places. *Acinetobacter* was the second most frequently identified genus in this study, and it has been reported to be isolated from moist skin and in a variety of foodstuffs. This bacterial genus is known to be resistant to dry conditions, resulting in survival in various environmental conditions (Rosenberg et al., 2013). In addition, we found that contamination of *Enterobacteriaceae* was observed from the outdoor air and the phyllosphere to the apron, fan, and pre-preparation area. *Enterobacteriaceae* also directly cross-contaminated from the food to the final preparation area. *Enterobacteriaceae* mostly belong to spoilage bacteria and can remain after cleaning and disinfection with a strong biofilm formation, suggesting that *Enterobacteriaceae* might be another significant risk factor for cross-contamination (Stellato et al., 2015b; Wang et al., 2017).

Non-food contact surfaces such as apron and fan exhibited high concentrations of bacteria, although the samples were obtained after cleaning and sanitizing the kitchen. These results are supported by a previous work that the fan had the most diverse microbiota, potentially from the adherence of the microorganisms through aerosol transmission (Chiller et al., 2001). Furthermore, our results revealed that microbial contamination in the fan was strongly linked to the outdoor air in all quarters, and the contamination route of *Streptophyta* was observed between the fan and other surface types. *Streptophyta* spp. have been frequently found in outdoor air, floor dust, and hair samples (Costello et al., 2009; Hospodsky et al., 2012; Kembel et al., 2012; Adams et al., 2015). In terms of the *Streptophyta* migration pathway, it has been reported that *Streptophyta* enters indoor environments from the outside air in the form of dust, or the interior by a person’s clothes, skin, and hair in the form of particles. When *Streptophyta* enters from outside air, the *Streptophyta* attach to the fan and spread to other surfaces, causing subsequent contamination (Hospodsky et al., 2012). Therefore, it is necessary to be alert to the use of fans in food manufacturing environments.

*Corynebacterium, Propionibacterium*, *Staphylococcus*, and lactic acid bacteria such as *Lactobacillus, Weissella*, and *Lactococcus* have been extensively studied for food spoilage and their presence in human skin (Stellato et al., 2015a; Lloyd-Price et al., 2016). In this study, skin-originated bacteria were frequently observed on food contract surfaces as well as the pre-preparation area and the final preparation area, suggesting that unheated food such as fresh products can be contaminated through human skin. Moreover, our results showed that hand-oriented microbiota was observed on various surfaces, suggesting that workers’ hands can be critically connected to microbial contamination. Our results also showed that cooking conditions and/or spatial structure affected the proximate cluster of the microbial communities; the cooking area that was separated from other surface types showed less cross-contamination, and sinks and fans, cooking pots, and counter tops were grouped based on the adjacent location.

High throughput sequencing has recently been developed as a great source to provide the microbial communities in food processing facilities (Aravindraja et al., 2013). This techniques can provide more information than the culture dependent community analysis and apply to any food processing environment for mapping the contamination, biofilm and persistence (Lim et al., 2017; De Filippis et al., 2021). However, the sampling materials, the sampling methods such as swabbing or sponge, DNA extraction methods and the sequencing technology can significantly impact the result of the bacterial communities (Maillet et al., 2021). In addition, most culture-independent sequencing methods are based on the DNA extraction of the target samples which may contain dead bacteria. Therefore, standardized protocols and technology should be developed and applied.

This study was limited to one kitchen environment that may not be representative of most cafeteria kitchens. However, from 1 year of repetitive study, our results provided critical information regarding major contaminated microorganisms on surfaces in the food manufacturing environment, differentiated by season, and contamination routes of such microorganisms and the main sources of contamination. Therefore, the results of this study would be a practical reference for foodservice facility managers to maintain hygiene practices by identifying the sources of contamination during food manufacture. The biofilms caused by the bacterial species are another particular phenomenon in food processing facilities. There are several reports that multispecies-biofilms between microorganisms isolated from food manufacturing environments can increase biofilm forming ability or resistance to environmental stress (Kostaki et al., 2012; Jahid et al., 2015; Røder et al., 2015; Sanchez-Vizuete et al., 2015). Therefore, this study could be a useful source for food risk control to understand interactions between microorganisms from food contact surfaces and foodborne pathogens, which have been frequent problems in foodservice.

## Conclusion

Indigenous bacteria that are present in raw ingredients and processed foods as well as on workers’ hands are introduced to the kitchen and then transferred to other food, employees, and equipment, or the bacteria can be removed by washing, and/or die during cooking or the sanitization process. However, if the kitchen is not cleaned and sanitized properly, significant cross-contamination risks from the survival of these bacteria exist. The kitchen contains bacteria that form biofilms, which are difficult to remove and facilitate the survival of foodborne pathogens. While contamination of bacteria is multifactorial and highly variable among individuals and cultures, our study can be beneficial to understand the biogeography of microorganisms in cooking areas and to map the transmission routes and cross-contamination sources during the cooking process.

## Data Availability Statement

The datasets presented in this study can be found in online repositories. The names of the repository/repositories and accession number(s) can be found in the article/Supplementary Material.

## Author Contributions

EL and JK performed the research and the data analysis. WS and OK designed the research study and supervised the project. EL, J-SK, BK, HK, and OK performed the literature review and wrote the manuscript. All authors have revised and approved the final version of the manuscript.

## Conflict of Interest

The authors declare that the research was conducted in the absence of any commercial or financial relationships that could be construed as a potential conflict of interest.

## Supplementary Material

Supplementary Figure 1 Rarefaction curve of sequence data.

Supplementary Figure 2 Alpha-diversity indices of 16S rRNA amplicons analyzed by quarters **(A)**, preparation areas **(B)**, and food-contact and non-food contact surfaces **(C)** of the kitchen. The dots are outliers, one asterisk mark mean *p* < 0.05 and two asterisks mean *p* < 0.01.

Supplementary Figure 3 Relative abundances of bacteria at the phylum **(A)** and genus **(B)** level in food-contact and non-food contact surfaces.

Supplementary Table 1 Summarized analyzed sequence data information.

Supplementary Table 2 List of source contribution of bacterial species.
