# Supplementary material for: Metagenomic Analysis of Microbial Composition Revealed Cross-Contamination Pathway of Bacteria at a Foodservice Facility
Source: Front Microbiol. 2021 Apr 12;12:636329. doi: 10.3389/fmicb.2021.636329 (PMC8071874; doi:10.3389/fmicb.2021.636329)
Supplement: Supplementary Figure 1 — Rarefaction curve of sequence data. [file Data_Sheet_1.docx]

# Supplementary Material

## Supplementary Figures

##
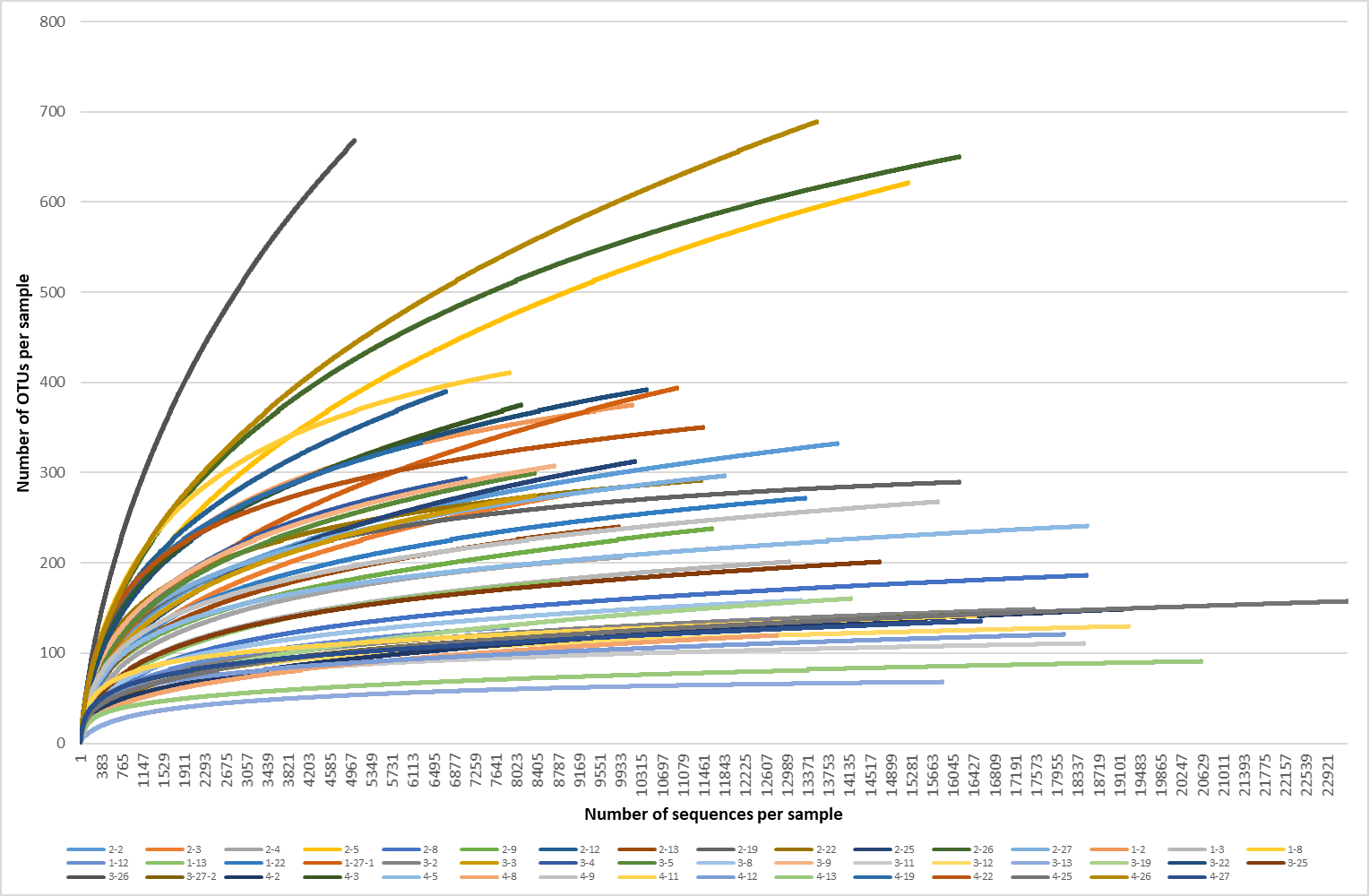


## Figure S1. Rarefaction curve of sequence data

##
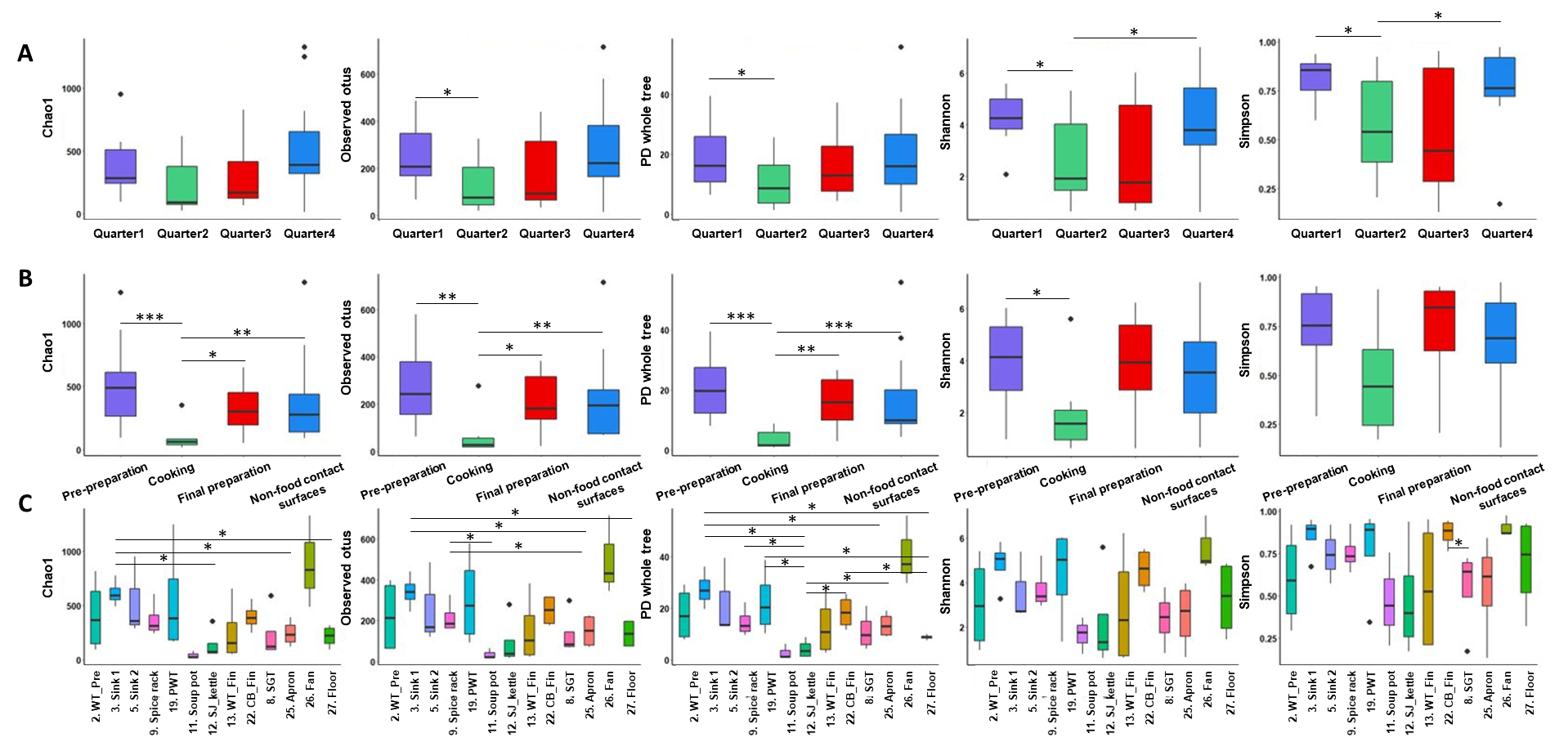


## Figure S2. Alpha-diversity indices of 16S rRNA amplicons analyzed by quarters (A), preparation areas (B) and food-contact and non-food contact surfaces (C) of the kitchen.


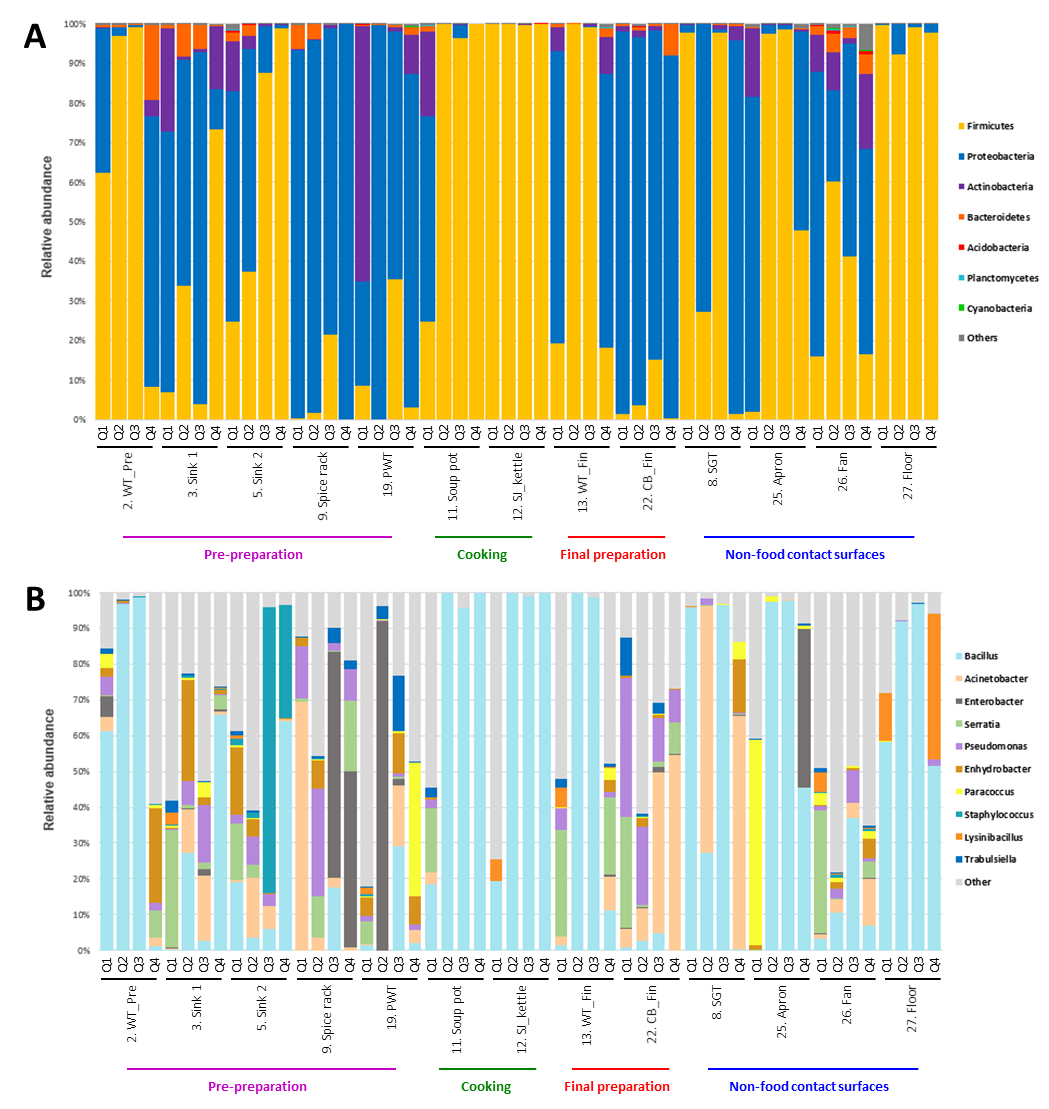


**Figure S3.** Relative abundances of bacteria at the phylum (A) and genus (B) level in food-contact and non-food contact surfaces.
